# Intramuscular oxytocin versus intravenous oxytocin to prevent postpartum haemorrhage at vaginal delivery (LabOR trial): study protocol for a randomised controlled trial

**DOI:** 10.1186/s13063-017-2269-9

**Published:** 2017-11-15

**Authors:** Nita Adnan, Fiona Boland, Deirdre J. Murphy

**Affiliations:** 1 Academic Department of Obstetrics and Gynaecology, Trinity College, University of Dublin & Coombe Women & Infants University Hospital, Dublin 8, Ireland; 2 Department of General Practice, Royal College of Surgeons in Ireland, Dublin 2, Ireland

**Keywords:** Intramuscular oxytocin, Intravenous oxytocin, Postpartum haemorrhage, Active management third stage of labour, Randomised controlled trial

## Abstract

**Background:**

Primary postpartum haemorrhage (PPH) is one of the leading causes of maternal morbidity and mortality worldwide. The most common cause of primary PPH is uterine atony. Atonic PPH rates are increasing in developed countries despite routine active management of the third stage of labour. In less-developed countries, primary PPH remains the leading cause of maternal death.

Although the value of routine oxytocics in the third stage of labour has been well established, there is inconsistent practice in the choice of agent and route of administration. Oxytocin is the preferred agent because it has fewer side effects than other uterotonics with similar efficacy. It can be given intravenously or intramuscularly; however, to date, the most effective route of administering oxytocin has not been established.

**Methods/Design:**

A double-blind randomised controlled trial is planned. The aim of the study is to compare the effects of an intramuscular bolus of oxytocin (10 IU in 1 mL) and placebo intravenous injection (1 mL 0.9% saline given slowly) with an intravenous bolus of oxytocin (10 IU in 1 mL given slowly over 1 min) and placebo intramuscular injection (1 mL 0.9% saline) at vaginal delivery. The study will recruit 1000 women at term (>36 weeks) with singleton pregnancies who are aiming for a vaginal delivery. The primary outcome will be PPH (measured blood loss ≥ 500 mL). A study involving 1000 women will have 80% power at the 5% two-sided alpha level, to detect differences in the proportion of patients with measured blood loss > 500 ml of 10% vs 5%.

**Discussion:**

Given the increasing trends of atonic PPH it is both important and timely that we evaluate the most effective route of oxytocin administration for the management of the third stage of labour. To date, there has been limited research comparing the efficacy of intramuscular oxytocin vs intravenous oxytocin for the third stage of labour.

**Trial registration:**

ISRCTN Registry, ISRCTN14718882. Registered on 4 January 2016. Pilot commenced 12.12.2015; trial commenced 04.01.2016. The protocol (Ref 012012) was approved by the National Maternity Hospital Research Ethics Committee on 10.06.2015 and the Research Ethics Committee of the Coombe Women & Infants University Hospital (Ref 26-2015) on 09.12.2015.

**Electronic supplementary material:**

The online version of this article (doi:10.1186/s13063-017-2269-9) contains supplementary material, which is available to authorized users.

## Background

Primary postpartum haemorrhage (PPH) is one of the leading causes of maternal mortality worldwide [1]. Obstetric complications are increasing with the changing profile of labouring women, who are now more likely to be older, to be obese or to have significant medical co-morbidities [2, 3]. PPH-associated morbidity includes maternal exhaustion, difficulty breast feeding, anaemia, blood transfusion and the risks associated with receiving donor blood products. In severe cases it may lead to emergency surgery, admission to an intensive care unit or death [3, 4].

Recent estimates of global maternal mortality indicate that over 300,000 women, mostly from developing countries, lose their lives during pregnancy and childbirth every year [4]. PPH accounts for nearly one-quarter of maternal deaths worldwide [5]. The most common underlying cause of PPH is uterine atony [6]. PPH rates have been reported to be increasing in developed countries over the past 15 years including Australia, Canada and the USA [4]. A population-based cohort study conducted in Ireland reported trends in atonic postpartum haemorrhage over 11 years [7]. It was reported that the overall PPH rate among hospital-based deliveries has increased substantially from 1.5% in 1999 to 4.9% in 2009. The increase in atonic PPH rates were observed across vaginal and instrumental deliveries, as well as Caesarean deliveries. Importantly, there was a significant increase in the incidence of women needing blood transfusion, signalling a rise in more severe atonic PPH, unlikely to relate to prior under-reporting. The study authors advised further evaluation of the active management of the third stage of labour.

The value of routine oxytocics in the third stage of vaginal birth has been well established although the choice of agent and route of administration remain controversial. There are several uterotonics that can be used in the active management of the third stage of labour such as synthetic oxytocin, ergometrine, oxytocin-ergometrine, misoprostol and carbetocin. Several meta-analyses have been performed comparing these agents. Oxytocin is the preferred agent because it has fewer side effects compared with other uterotonics with similar efficacy [8, 9]. Oxytocin reduces the risk of PPH by about 60% and the need for therapeutic oxytocics by about 50% [10].

Oxytocin can be given intravenously or intramuscularly [11]. The intramuscular (IM) route has the advantage of ease of administration and requires relatively less skill to administer. Following IM injection, the effect on the uterus appears within 3–7 min and persists for 30–60 min. With the intravenous (IV) route the uterine response is almost instantaneous, within 1 min or less, compared to IM administration which results in a slower onset of action but produces a longer lasting effect [12, 13]. It is recognised that early rapid delivery of the uterotonic drug may be associated with a lower risk of postpartum haemorrhage; however, this may increase the risk of cardiovascular side effects [1, 14]. IV administration is the preferred route at Caesarean section where the risk of haemorrhage is higher.

A recent Cochrane systematic review [15] looked at the effect of prophylactic oxytocin for the management of the third stage of labour and found that it is effective in preventing blood loss ≥ 500 mL at any dose, in the range of 3–10 IU, compared with placebo. The need for additional uterotonics is reduced when a 10 IU dose is used and when given as an IV bolus. The guidelines of the Royal College of Obstetricians and Gynaecologists (RCOG) and the Royal College of Physicians of Ireland (RCPI) recommend an IM bolus dose of 10 IU of oxytocin following delivery of the infant [8, 9]. The WHO guidelines recommend oxytocin 10 IU intramuscularly or by slow IV injection [6].

In the Coombe Women & Infants’ University Hospital, both methods of administering oxytocin are commonly used. The traditional practice over several decades was to administer oxytocin 10 IU intravenously following delivery of the anterior shoulder of the baby. A new protocol was implemented recommending the IM route of oxytocin 10 IU in keeping with RCOG guidelines. Concerns were raised by established practitioners about a higher risk of haemorrhage with the IM route, which led to variation in practice. This presented the ideal opportunity for a randomised controlled trial (RCT). We chose to compare oxytocin 10 IU administered by IV vs IM injection in accordance with international prevention of PPH guidelines [6, 8]. Various trials have compared IV oxytocin slow bolus with placebo and with alternative uterotonics with no undesirable side effects [15, 16].

Early clamping of the umbilical cord was one of the first routine medical interventions in labour and its role in current practice has been questioned. Weeks, in an editorial, describes how its place in modern births was guaranteed by its incorporation into the triad of interventions that make up the traditional active management of the third stage of labour [17]. The earliest references are clear about the other two components of active management—oxytocin to contract the uterus and controlled cord traction to prevent retention of the placenta [1]. However, early cord clamping had no specific rationale and Weeks speculates that it probably entered the protocol by default because it was already part of standard practice. When this package was shown to reduce postpartum haemorrhage in the 1980s early cord clamping became enshrined in the modern management of labour. There are now very strong arguments to support deferred cord clamping for 1–3 min and this has resulted in an amendment to that effect in the RCOG and WHO Postpartum Haemorrhage Prevention guidelines [6, 8]. Deferred cord clamping was implemented as part of the current study protocol.

### Literature review

A search of Medline from 1965 to 2016 and of the Cochrane Library was undertaken for relevant systematic reviews, meta-analyses, RCTs and other clinical studies. The date of the last search was November 2016. There were no changes since the time of the last search during the finalisation of this study protocol. We intend to update this before publishing the results of the trial. The main keywords used were: IV oxytocin, IM oxytocin, active management of the third stage of labour, prevention of postpartum haemorrhage in vaginal delivery, prophylactic oxytocin in vaginal delivery, blood loss and uterotonic agent.

A recent Cochrane systematic review [18] identified a lack of a RCT that compares IM oxytocin with IV oxytocin for prevention of haemorrhage in vaginal delivery. At the time of drafting the LabOR trial protocol and commencing the study, there were no published RCTs that compare the effect of the two routes of oxytocin administration on blood loss ≥ 500 mL at vaginal delivery.

We have updated our literature review in preparation for publication of the study protocol and identified two recent RCTs that compare the IM and IV route of oxytocin for preventing haemorrhage in the third stage of labour. Dagderiven et al. compared oxytocin 10 IU intravenously in 1 L normal saline infused at a rate of 1 mL/min to oxytocin 10 IU intramuscularly. Blinding of the clinicians, participants and researcher were not mentioned in the study. Orhan et al. compared 10 IU oxytocin IV at 1 mL/min to oxytocin 10 IU IM. The birth attendants and participants were not blinded to the form of oxytocin administration. The research team who performed the measurement of blood loss were blinded. The two studies had 128 and 150 participants in each group, respectively, and neither study included the recommendation for delayed cord clamping (Table 1).

**Table 1:** Studies comparing the effect of IM oxytocin and IV oxytocin on haemorrhage after vaginal delivery

Author, citation	Study design	Exposures	Outcome measures	Results	Conclusions
Dagdeviren H et al. Arch Gynecol Obstet. 2016;294:911–916	RCT 256 women	10 IU IM vs 10 IU in 1 L normal saline IV 1 mL/min infusion after delivery of anterior shoulder	Measured blood loss and occurrence of 1° PPH	No differences in outcomes	Mode of oxytocin administration no significant differences on postpartum blood loss
E Orhan et al. Int J Gynecol. 2014;127:175–179	RCT 600 women 150 in each group Group IV divided into IV(A) and (B) Group IM divided into IM(A) and (B)	Group IV- 10 IU oxytocin 1 mL/min Group IM -10 IU oxytocin (A) after delivery of fetus (B) at delivery of anterior shoulder	Measured blood loss Δ Hb + Hct Side effects	Significant differences in Δ Hb + Hct in IV(A)group Blood loss slightly lower in this group but not significant difference	Mode and timing of administering oxytocin has no significant effects on blood loss but early IV administration may have beneficial effects

### Survey of current practice

Before undertaking this study, it was important to establish the need for such a trial. A survey was carried out in the UK in the form of a questionnaire looking at current practice during the third stage of labour [19]. The questionnaire was sent to fellows and members of the RCOG and to members of the RCM. The questionnaire focused on the frequency of usage of the active management, timing of administration of prophylactic uterotonic agents, type and route of administering uterotonics, timing of clamping the cord (at term and preterm births) and the use of controlled cord traction. The clinicians were also asked if they thought more evidence from RCTs was needed to guide care during the third stage of labour.

A total of 4630 questionnaires were sent. Of these 53% of the fellows and members of the RCOG responded and 71% of midwives responded. Of the respondents 93% of obstetricians and 73% of midwives reported ‘always or usually’ using active management of the third stage of labour for vaginal births. Syntometrine was the uterotonic agent ‘usually’ used at vaginal births by 86% of midwives and 79% of obstetricians; 89% of midwives and 81% of obstetricians thought more evidence from RCTs was needed to guide care during the third stage.

The findings of the survey show that active management of the third stage of labour is widely used by obstetricians and midwives in the UK, which is consistent with recommendations by NICE, WHO, Canada and the RCOG [1, 20, 21]. Syntometrine is the most commonly used uterotonic which was a surprise finding given that NICE, WHO and the RCOG guidelines clearly recommend use of oxytocin as the prophylactic uterotonic agent for the third stage. The NICE guideline recommends the IM route for oxytocin while WHO and RCOG guidelines mention both IM and IV oxytocin can be used. Most obstetricians and midwives thought more evidence from RCTs was needed to guide care in the third stage of labour.

### Aims and objectives

The aim of this study is to compare the effects of an intramuscular bolus of oxytocin (10 IU in 1 mL) and placebo IV injection (1 mL 0.9% saline given slowly) with an IV bolus of oxytocin (10 IU in 1 mL given slowly over 1 min) and placebo IM injection (1 mL 0.9% saline) at vaginal delivery in a double-blind RCT. The hypothesis is that the IV route will be associated with a lower incidence of PPH, but this may be at the expense of a higher incidence of side effects.

Specific objectives are listed below.

#### Primary outcomes

- To compare the incidence of PPH (> 500 mL measured blood loss) following IM oxytocin vs IV oxytocin.

#### Secondary outcomes

- To compare the incidence of side effects following administration of oxytocin (headache, vomiting, hypotension, tachycardia);
- to compare the estimated mean blood loss and early lochial loss following IM oxytocin compared with IV oxytocin;
- to compare the incidence of major obstetric haemorrhage (measured blood loss ≥ 1000 mL);
- to compare the objective change in haemoglobin and haematocrit before and 24 h after delivery between the two groups;
- to compare the incidence of severe anaemia (Hb fall ≥ 20%) 24 h after delivery between the two groups (or patient transfused);
- to compare the need for blood transfusion and/or blood products;
- to compare the need for an additional uterotonic agent between the two groups;
- to compare the postnatal length of stay in labour ward/HDU and in the hospital.

## Methods/Design

**Fig. 1:**
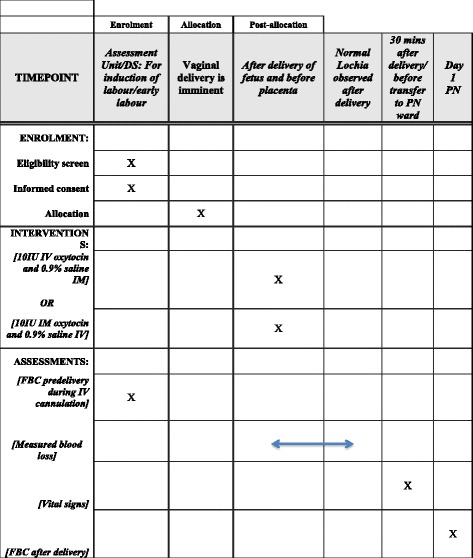
LabOR Trial schedule of enrolment, interventions and assessments

A double-blind RCT is proposed to be carried out in the Coombe Women & Infants University Hospital (Fig. 1).

### Recruitment and allocation

#### Research participants

##### Inclusion criteria

The study will be limited to women at term (> 36 weeks) with singleton pregnancies who are aiming for a vaginal delivery.

##### Exclusion criteria

Women will be excluded if they have requested physiological management of the third stage of labour or if there has been a prior decision to use an additional oxytocin infusion by the woman’s care giver because of an increased risk of postpartum haemorrhage. Women who are deemed at increased risk of postpartum haemorrhage are those with a previous history of postpartum haemorrhage due to atonic uterus, known fibroids, a history of coagulopathy receiving anti-coagulant therapy and those who have thrombocytopenia. Women with pre-existing cardiovascular disease will be excluded as well as women who do not understand English and women under the age of 18 years.

#### Method of assigning treatment groups

##### Randomisation

Randomisation will be performed using an automated web-based randomisation system hosted by the Nottingham Clinical Trials Unit, a UKCRC-registered trials unit thereby ensuring concealment. Allocation will be stratified by parity (nulliparous/multiparous), and blocked using random permuted blocks of varying size.

##### Blinding

Following randomisation and allocation, the research fellow will draw up the medication and placebo into two separate sterile 2-mL syringes. This will be checked by the midwife in charge of the woman’s care in the labour room, in keeping with the hospital policy of drawing up medications. The randomisation and labelling of the syringes will occur outside the room. The syringes will be labelled as Drug 1-IM or Drug 2-IV injection and will be returned to the midwife in the delivery room. This will ensure blinding of everyone except the research fellow who will have no role in the management of the patient. Having the research fellow blinded is not possible for three reasons. First, it is not possible to pre-prepare syringes due to a lack of stability data for pre-prepared oxytocin syringes. Second, it has not been possible to obtain identically matched placebo ampoules. Third, the research ethics committees required that the midwife caring for the patient observes the drawing up of the oxytocin and placebo so that she has verified the contents of the injections she will be administering, albeit blinded to which syringe contains the active agent.

### Intervention

Women will be informed of the study antenatally in the outpatient clinics and antenatal classes; they will be provided with an information leaflet which has been approved by the Research Ethics Committee in National Maternity Hospital by the research fellow. Formal recruitment and written consent will take place when women attend the assessment unit with symptoms and signs of early labour or when they are admitted for induction. The admitting midwife will approach potentially eligible women and establish whether they are suitable for recruitment. A research fellow/midwife will approach eligible women and seek written informed consent (see ‘Screening procedure’). Following written informed consent, the chart will be labelled with a trial sticker in order to inform the participants’ carers, i.e. midwives and obstetricians, that she is participating in the study.

When the woman is confirmed to be in labour or is ready to have her labour induced, she will be brought into a room in the delivery suite. When a cannula is being sited (e.g. for epidural, IV fluid or syntocinon infusion, which is common practice) a sample will be taken for a full blood count at the same time. Randomised allocation will take place when the second stage of labour has been diagnosed in nulliparous women. For multiparous women, random allocation will take place when they have been diagnosed in active labour and vaginal delivery is considered imminent. Following randomisation, the woman will be allocated to receive either IM oxytocin 10 IU and a placebo (1 mL 0.9% normal saline) intravenously or IV oxytocin 10 IU slow bolus over 1 min and a placebo (1 mL 0.9% normal saline) intramuscularly.

The syntocinon and the normal saline ampoules to be used in the trial are currently being used routinely in the delivery suite in the Coombe Women & Infants University Hospital.

Name of the medicinal product: syntocinon 10 IU/mL solution for infusion or injection. Marketing authorisation holder: Sigma-Tau Industrie Farmaceutiche.

Name of placebo: sodium chloride 0.9% w/v injection BP. Marketing authorisation holder: Noridem Enterprises Ltd.

The research team will draw up the medication and placebo into two separate sterile 2-mL syringes. This will be checked by the midwife in charge of the woman’s care in the labour room, in keeping with the hospital policy of drawing up medications. The randomisation and labelling of the syringes will occur outside the room. The syringes will be labelled as Drug 1-IM or Drug 2-IV injection.

The syringes which are labelled Drug 1-IM and Drug 2-IV will be given to the midwife who is in charge of the woman’s care in the labour ward. Once the baby is delivered, the IM syringe will be administered first followed by the IV syringe. Both the midwife and the labouring woman will be blind to the treatment allocation. The researcher who has labelled the syringes plays no role in the patient’s care or in the ascertainment or recording of outcome data.

In order to overcome potential variations in the actual practice of active management of the third stage of labour, a working definition has been specified.

#### Experimental arm: intravenous oxytocin and placebo

##### Uterotonic agent

Immediately following delivery of the baby and preferably within 1 min of birth, 1 mL 0.9% normal saline is given intramuscularly into the thigh muscle followed by oxytocin 10 IU given slowly intravenously over 1 min through a cannula [22].

##### Deferred cord clamping

The umbilical cord will be clamped at approximately 1–3 min after the birth of the baby. This is in keeping with the current recommendations from the WHO and RCOG postpartum haemorrhage guideline [6, 8]. The baby can be positioned either on the mother’s chest or abdomen following the birth while the cord is intact and immediate newborn care can be commenced at the same time. The baby should not be raised more than this level with the cord still intact due to the effects of gravity on placental transfusion [23]. The cord will be clamped close to the umbilicus in the usual manner and the timing of cord clamping will be recorded in the patient’s medical notes and case report form (CRF).

However, early cord clamping may be necessary in situations of emergency such as postpartum haemorrhage, presence of a tight nuchal cord or if immediate resuscitation is required for an asphyxiated baby. In situations as these, where expedient management is important to minimise complications to the patient or the baby, the obstetrician or midwives who are attending the birth should decide on the timing of clamping the cord, which will be recorded in the patient’s medical notes with the reason for early cord clamping.

##### Placental delivery

The placenta should be delivered by controlled cord traction following cord clamping and cutting, once the signs of separation are apparent (uterine contraction, show of blood and apparent cord lengthening). The cord will be pulled gently while applying counter traction to the uterus with the other hand (Brandt–Andrews manoeuvre). The placenta should be held in two hands and rotated, careful to ensure the membranes do not tear.

#### Standard arm: intramuscular oxytocin and placebo

##### Uterotonic agent

Immediately following delivery of the baby and preferably within 1 min of birth, oxytocin 10 IU is given intramuscularly into the thigh muscle and 1 mL 0.9% normal saline is given intravenously through the cannula.

The steps involving umbilical cord clamping and placental delivery are the same as in the experimental arm.

The two interventions only differ in the route of administering oxytocin and the placebo.

Blood loss following vaginal deliveries will be measured by direct collection of blood and the gravimetric method.

For women who have a normal vaginal delivery, a fresh delivery sheet is placed under her buttocks as soon as the baby is born and placed on the woman’s chest/abdomen. Once there is no concern of further active bleeding, the blood loss will be estimated by weighing all the soiled materials (swabs, pads, disposable sheets) on a scale and subtracting the known weights of these materials to determine the blood loss. Women remain on the labour ward for a minimum of 1 h after the birth and are monitored throughout that time period. They are then transferred to the postnatal ward if there are no apparent complications.

For women who have an assisted vaginal delivery, Comfy Guard®, a surgical drape with a pouch for collecting vaginal blood loss, will be placed under the woman’s buttocks in preparation for birth and before delivery of the placenta. The bag is transparent allowing continuous monitoring of blood loss. The bag will be left in situ until the birth attendant is no longer concerned about blood loss, such as when a sanitary towel is applied to the vulva. The blood collected in the surgical drape will be weighed. Blood-soaked swabs and additional drapes will also be weighed and the known dry weight of the swabs and drapes will be subtracted. This volume will be added to the measured blood volume from the surgical drape.

Any delayed blood loss occurring within 24 h of delivery will also be recorded. It is possible that there may be a difference in estimates of blood loss based on different durations of blood loss collection for women with early or delayed transfer to the postnatal ward. This potential limitation will be addressed by routinely documenting any additional significant blood loss within 24 h of delivery.

Maternal vital signs will be recorded after delivery and before transfer to the postnatal ward. The occurrence of side effects will be recorded using an observation form (attached with the application). Deviations from the standard procedure will be recorded. Should the uterus remain atonic despite the trial intervention, the obstetrician/midwife/anaesthetist can use any additional uterotonic agent in the usual way according to the hospital postpartum haemorrhage protocol.

#### Follow-up

A full blood count will be performed at day 1 (24 h) after delivery to assess haemoglobin and haematocrit. Clinical follow-up of the mother will be completed before hospital discharge.

#### Outcome measures

The primary outcome measures are PPH (measured blood loss > 500 mL). Measured blood loss of ≥ 500 mL has been chosen as a clinically relevant outcome and to allow direct comparisons with other studies. It is widely accepted that clinicians underestimate rather than overestimate blood loss and for this study we will formally measure blood loss rather than relying on subjective estimates.

Secondary outcomes include the incidence of side effects following administration of oxytocin, mean estimated blood loss, major obstetric haemorrhage (measured loss ≥ 1000 mL), change in haemoglobin and haematocrit, significant anaemia (Hb fall ≥ 20%) 24 h after delivery, need for blood transfusion, length of postnatal stay in hospital (and specifically admission for high-dependency care) and the need for an additional uterotonic agent. Hypotension will be defined as a fall in blood pressure > 30% below the pre-delivery blood pressure and/or use of ephedrine and tachycardia will be defined as a persistent heart rate of above 100 bpm.

#### Definition of end-of-trial

The trial will be considered complete after the final review of the last trial participant. Trial completion will be notified to the Competent Authority and the Ethics Committees using the appropriate form.

#### Withdrawal of research participants from study protocol

Women who previously agreed to participate in the trial can voluntarily withdraw their consent and they are not obliged to give a reason for their withdrawal. This is written in the research participant’s information leaflet and will be explained during the consenting procedure.

Women will be withdrawn from the trial if their primary care giver has decided on the preferred route of oxytocin administration for the third stage.

The reason for withdrawal will be recorded in the CRF of the trial and the management for the women who are withdrawn from the trial will follow routine hospital care or as specified by the primary clinician caring for them.

#### Statistical analysis

**Fig. 2:**
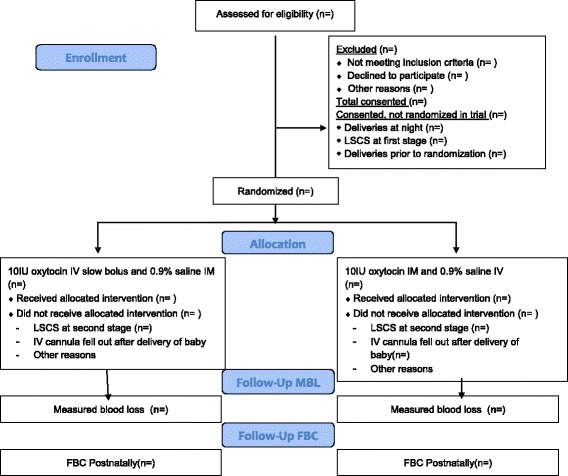
CONSORT *flowchart*

Data analysis and reporting will proceed according to CONSORT guidelines [24] for RCTs and will be conducted blinded to group status by the trial statistician and researcher (Fig. 2). The first stage of analysis will be to use descriptive statistics to describe recruited individuals in relation to those eligible and to investigate comparability of the trial arms at baseline. The primary analyses will involve intention-to-treat comparisons between the two groups for the primary outcomes, with transformation as appropriate after examination of distributions and adjusted for stratification variables (i.e. parity). The study aims to evaluate haemorrhage at vaginal delivery and women who deliver by Caesarean section will be excluded post-randomisation and accounted for in the CONSORT diagram. Secondary analyses will investigate the effects of further adjustment for any variables displaying marked imbalance between the arms at baseline. Secondary outcomes will be analysed in a similar way. All analyses will use appropriate (that is, logistic or linear) regression models, with results presented as point estimates (odds ratios or difference in means), 95% confidence intervals and *p* values. Further secondary analyses will involve planned subgroup analyses and will use multivariable regression models with appropriate interaction terms to ascertain any differential effects in relation to induction of labour, parity and history of previous postpartum haemorrhage. The SPIRIT Checklist is provided as Additional file 1.

#### Sample size

We estimate from the Coombe Hospital annual report in 2010 and a retrospective audit of labour ward deliveries that 6–10% of study participants will have an estimated blood loss of > 500 mL. These estimates are based on observed recording of haemorrhage and it is well recognised that obstetricians and midwives underestimate blood loss. It is therefore likely that the rate of postpartum haemorrhage based on measured blood loss will be higher, consistent with our findings on the ECSSIT trial [25].

With 500 patients per group for analysis, the study will have 80% power at the 5% two-sided alpha level, to detect differences in the proportion of patients with measured blood loss > 500 mL of 10% vs 5% or 15% vs 9%, equivalent to odds ratios of 0.50 and 0.55, respectively. It could be argued that any difference in effect on the primary clinical outcome would be worth detecting. Rather, given the need for timely delivery of evidence, we have specified detectable differences for realistic sample sizes recruited within a reasonable time frame within the constraints of the available funding. The results can be included in future Cochrane systematic reviews and may support funding of a future larger trial if smaller but clinically relevant differences are detected.

Over the course of 18 months we aim to recruit a minimum of 1000 patients representing 20% of the available population; this conservative estimate takes account of eligibility criteria and non-English speaking women. The final sample size will be slightly inflated to allow for women who subsequently deliver by Caesarean section and for missing outcome data (blood loss not measured or full blood count not taken at the correct time).

#### Timetable

Total 24 months: commenced 1 January 2016; estimated completion date 30 December 2017.

Months 0–3: validate datasets; raise study awareness; finalise recruitment procedures.

Months 4–21: recruit participants and complete documentation and follow-up.

Months 22–24: analysis; final report; peer-review publications; presentations.

#### Governance issues

##### Data management

Data will be collected in duplicate on a CRF at the time of recruitment by a trained researcher. The researcher will also be responsible for ensuring that the details of the delivery are recorded and the blood loss measured. The inpatient notes will be marked so that they can easily be recovered following discharge from hospital. After discharge, the CRF will be collected by the local coordinator and the completeness of the data checked against the woman’s notes. Any errors will be followed up at this time. The data will be entered into a computer database (password-protected) within a physically secure office in the Coombe Women & Infants University Hospital. Regular backups will be carried out to protect from risk of data loss.

##### Trial management group

This group will be in charge of the everyday running of the trial. The full group will meet four-monthly and as required. Day-to-day decision-making will be by Professor Murphy and the trial researcher.

##### Trial steering committee (TSC)

A TSC will be set up which will have overall supervision of the trial. It will meet before commencement of the trial and then at least six-monthly until completion. A meeting of the TSC will be held within one month of every data monitoring and ethics committee (DMEC) meeting to consider their recommendations. An independent Chair has been confirmed for the TSC (Dr C Lynch, Consultant Obstetrician and sub-specialist in Maternal-Fetal Medicine).

##### Data monitoring and ethics committee

An independent safety and data monitoring committee will also be formed. They will meet yearly to examine recruitment figures, baseline data, retention and adverse events. The DMEC will not undertake any formal interim analyses for either safety or effectiveness and therefore there are no formal stopping rules. The two regimens under evaluation have been used routinely in the study centre. However, all adverse events will be reported to the DMEC who will report these data to the TSC. Additionally, in the event of a postpartum haemorrhage, clinicians will be free to manage the patient according to the hospital postpartum haemorrhage protocol or as dictated by the lead clinician in keeping with the clinical circumstances.

##### Safety considerations

Serious or unexpected serious adverse reactions (SUSARs) will be recorded and reported using the Health Products Regulatory Authority (HPRA)-approved online report form and hard copy form. SUSARs include maternal death, emergency surgery (other than Caesarean section) in the two weeks following randomisation, transfusion of over 4 units of blood, admission to intensive care unit or suspected drug reactions. In the event of a SUSAR, the report form will be completed by the local trial co-ordinator and faxed to the trial coordinating centre at the Coombe Women’s Hospital. The sponsor will report the SUSARs to the HPRA and the Ethics Committee. Fatal SUSARs will be reported within seven days and non-fatal SUSARs will be reported within 15 days [26].

## Discussion

### Potential and implementation of the findings

It is both important and timely that we evaluate the optimal management of the third stage of labour, taking account of changes in practice relating to delayed cord clamping. Rates of postpartum haemorrhage have risen over the last two decades and continue to rise. Safe delivery and postnatal wellbeing are a priority for pregnant women. Obstetricians, obstetric anaesthetists, midwives and pregnant women need high quality evidence on which to base management approaches. The optimal approach to use of oxytocin in the third stage of labour is a poorly evaluated component of maternity care, despite its widespread use. The overall aim of this project is to reduce maternal haemorrhagic morbidity and its attendant risks at vaginal delivery. In addition, we will address the economic implications of the two approaches being evaluated.

### Dissemination

We aim to raise awareness of the clinical question and our proposed research approach at both local and national meetings. A final report will be prepared for the funding body and papers will be prepared for peer-review publication and national/international dissemination. The PI has been involved in national and international guideline development and in the publication of Cochrane systematic reviews. The aim will be to update these important resources in order to disseminate and implement evidence-based practice as widely as possible. The greatest potential impact of the findings is in low and lowest-income settings and we will interact with the WHO guideline group who produce clinical practice guidelines on a global basis.

#### Trial status

The trial is ongoing.

## Additional file

Additional file 1: SPIRIT 2013 checklist. (DOC 122 kb)

## Abbreviations

CONSORT: Consolidated Standards of Reporting Trials
CRF: Case report form
DMEC: Data monitoring and ethics committee
DS: Delivery suite
ECSSIT: Elective Caesarean Section Syntocinon Infusion Trial
Hb: Haemoglobin
HDU: High Dependency Unit
HPRA: Health Products Regulatory Authority
IM: Intramuscular
IV: Intravenous
LabOR trial: Labour Oxytocin Randomised Trial
NICE: National Institute for Health and Care Excellence
PI: Principal investigator
PN: Postnatal
PPH: postpartum haemorrhage
RCM: Royal College of Midwives
RCOG: Royal College of Obstetricians and Gynaecology
RCPI: Royal College of Physicians Ireland
RCT: Randomised controlled trial
SUSARs: Serious or unexpected serious adverse reactions
TSC: Trial steering committee
UKCRC: UK Clinical Research Collaboration
WHO: World Health Organization
